# Comparative genomics reveals distinct host-interacting traits of three major human-associated propionibacteria

**DOI:** 10.1186/1471-2164-14-640

**Published:** 2013-09-22

**Authors:** Tim N Mak, Monika Schmid, Elzbieta Brzuszkiewicz, Guanghong Zeng, Rikke Meyer, Karen S Sfanos, Volker Brinkmann, Thomas F Meyer, Holger Brüggemann

**Affiliations:** 1 Department of Biomedicine, Aarhus University, Aarhus, Denmark; 2 Proteomics Core Facility, Max Planck Institute for Infection Biology, Berlin, Germany; 3 Institute of Microbiology and Genetics, Georg August University Goettingen, Goettingen, Germany; 4 Interdisciplinary Nanoscience Center (iNANO), Aarhus University, Aarhus, Denmark; 5 Department of Pathology, Johns Hopkins University School of Medicine, Baltimore, MD, USA; 6 Microscopy Core Facility, Max Planck Institute for Infection Biology, Berlin, Germany; 7 Department of Molecular Biology, Max Planck Institute for Infection Biology, Berlin, Germany

**Keywords:** Cutaneous propionibacteria, *Propionibacterium acnes*, *Propionibacterium granulosum*, *Propionibacterium avidum*, Exopolysaccharide, Pilus/pili, Surfome

## Abstract

**Background:**

Propionibacteria are part of the human microbiota. Many studies have addressed the predominant colonizer of sebaceous follicles of the skin, *Propionibacterium acnes*, and investigated its association with the skin disorder acne vulgaris, and lately with prostate cancer. Much less is known about two other propionibacterial species frequently found on human tissue sites, *Propionibacterium granulosum* and *Propionibacterium avidum*. Here we analyzed two and three genomes of *P. granulosum* and *P. avidum*, respectively, and compared them to two genomes of *P. acnes*; we further highlight differences among the three cutaneous species with proteomic and microscopy approaches.

**Results:**

Electron and atomic force microscopy revealed an exopolysaccharide (EPS)-like structure surrounding *P. avidum* cells, that is absent in *P. acnes* and *P. granulosum*. In contrast, *P. granulosum* possesses pili-like appendices, which was confirmed by surface proteome analysis. The corresponding genes were identified; they are clustered with genes encoding sortases. Both, *P. granulosum* and *P. avidum* lack surface or secreted proteins for predicted host-interacting factors of *P. acnes*, including several CAMP factors, sialidases, dermatan-sulphate adhesins, hyaluronidase and a SH3 domain-containing lipoprotein; accordingly, only *P. acnes* exhibits neuraminidase and hyaluronidase activities. These functions are encoded on previously unrecognized island-like regions in the genome of *P. acnes*.

**Conclusions:**

Despite their omnipresence on human skin little is known about the role of cutaneous propionibacteria. All three species are associated with a variety of diseases, including postoperative and device-related abscesses and infections. We showed that the three organisms have evolved distinct features to interact with their human host. Whereas *P. avidum* and *P. granulosum* produce an EPS-like surface structure and pili-like appendices, respectively, *P. acnes* possesses a number of unique surface-exposed proteins with host-interacting properties. The different surface properties of the three cutaneous propionibacteria are likely to determine their colonizing ability and pathogenic potential on the skin and at non-skin sites.

## Background

The genus *Propionibacterium* belongs to the phylum *Actinobacteria* and contains classical (or dairy) and cutaneous species. Whereas classical species such as *Propionibacterium freudenreichii* are considered to have probiotic effects [1] and are rather well characterized due to their importance in the dairy industry, cutaneous species are less well understood. The three most important cutaneous species are *P. acnes*, *P. avidum* and *P. granulosum*; these species can be found on the skin of virtually every human being, and they are also found on other tissue sites, including the gastrointestinal tract, lungs, and the prostate [2-6]. As part of the skin microbiota, both *P. acnes* and *P. granulosum* are found in sebaceous-rich areas, but *P. acnes* predominates in areas such as scalp, forehead, ear, back, and alae nasi [2,7]. *P. avidum* prefers moist rather than oily areas; it is found mainly in the anterior nares, axilla, and rectum [7].

The role of human-associated bacterial species belonging to the genus *Propionibacterium* is largely unknown; these species are described as commensals, saprophytes, parasites or opportunistic pathogens. The pathogenic side of cutaneous propionibacteria, in particular *P. acnes* is slowly gaining attention. Apart from its possible role in acne vulgaris due to its immunostimulatory property, *P. acnes* has been associated with a number of other diseases [8,9]. Recently, *P. acnes* were found in diseased prostatic tissue [5,6,10], and its contribution to prostate pathologies is currently under investigation. In our previous study, we isolated *P. acnes, P. avidum* and *P. granulosum* from radical prostatectomy specimens [10]. Little is known about the association of *P. avidum* and *P. granulosum* with human diseases. *P. avidum* has been found to cause abscess formation, in particular after surgical intervention; it has been described as the cause of abdominal wall and intra-peritoneal, perianal, psoas, splenic, and breast abscesses [11-13]. The disease association of *P. granulosum* is less clear, though it has been found in a few cases of endocarditis and endophthalmitis, and has been associated, like *P. acnes*, with sarcoidosis [14,15].

It is not understood if all cutaneous propionibacteria have similar disease-causing potentials. The genome sequence of *P. acnes* and subsequent studies have highlighted host-interacting factors such as CAMP factors, hemolysins, sialidases and dermatan-sulphate adhesins [16-20]. To date it is not clear if these factors are shared in all cutaneous propionibacteria. Here, we provide genomic insight into *P. avidum* and *P. granulosum*, and compare these genomes to *P. acnes*. We also performed electron microscopy and atomic force microscopy analysis on these species to further highlight differences among cutaneous propionibacteria. Together with proteomic data, our study highlights the individuality of each of the three human-associated propionibacterial species. In particular, the distinct surface structures suggest that each species interacts differently with the human host, which likely results in distinct pathogenic potentials.

## Results

### Comparative genome analysis of cutaneous propionibacteria

Seven genomes were analyzed and compared, two of each species, *P. acnes* (strains 266 and KPA) and *P. granulosum* (strains DSM20700 and TM11), and three genomes of *P. avidum* (44067, ATCC25577 and TM16). Four genomes were available from public databases (*P. acnes* KPA171202 (KPA) (GenBank: AE017283) [16], *P. acnes* 266 (GenBank: CP002409) [21], *P. avidum* 44067 (CP005287) [22] and *P. avidum* ATCC25577 (GenBank: NZ_AGBA00000000)) and we draft sequenced three additional ones (*P. granulosum* DSM20700 and TM11) and *P. avidum* TM16. *P. granulosum* TM11 and *P. avidum* TM16 were both isolated from radical prostatectomy specimens [10], and *P. avidum* 44067 was isolated from a human skin abscess [22]. P. *avidum* ATCC25577 and *P. granulosum* DSM20700 are both type strains.

**Figure 1:**
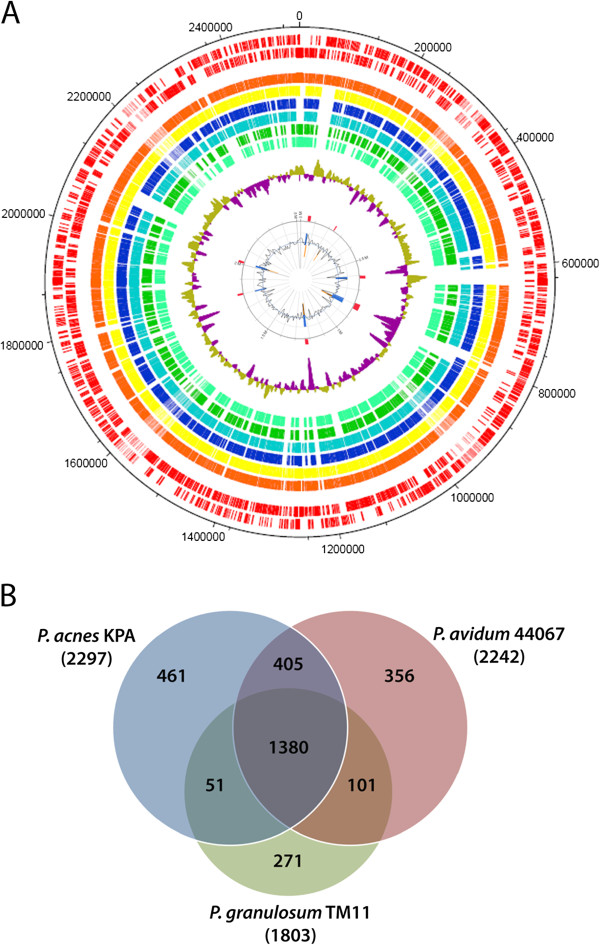
**Genome comparison of three propionibacteria. A)** All CDS of *P. avidum* 440677 (two red outer rings, representing CDS on plus and minus strand) were compared by bidirectional blast with all CDS of *P. avidum* ATCC25577 (orange) and *P. avidum* TM16 (yellow), *P. acnes* KPA (blue), *P. acnes* 266 (light blue), *P. granulosum* TM11 (green), and *P. granulosum* DSM20700 (marine). Gaps indicate the absence of CDS homologs of *P. avidum* 44067 in the respective genomes. The inner ring (in purple and olive) represents the G + C content distribution of the *P. avidum* 44067 genome (window size 10000 bp, step size 200 bp). The most inner circle depicts predicted islands (in red) possibly acquired by horizontal gene transfer (predictions from IslandViewer; results from two different algorithms are shown: orange, Sigi-HMM; blue, IslandPath-DIMOB). *P. avidum* harbors 7 genomic regions that are predicted to be horizontally acquired. Please see also Additional file 3 for the genome comparisons of *P. granulosum* TM11 and *P. acnes* KPA. **B)** A VENN diagram depicts the number of species-specific CDS and those shared by all three propionibacteria. The three-way comparison is based on bidirectional blast results (Additional file 2). 1380 CDS have homologs in all three propionibacteria (>25% amino acid identity), which represents 76%, 61% and 60% of all CDS in *P. granulosum* TM11*, P. avidum* 44067 and *P. acnes* KPA*,* respectively.

First analysis revealed the much smaller genomes of the two *P. granulosum* strains, which is in average 400 kb smaller than *P. acnes* and *P. avidum* (Additional file 1). A bidirectional Blast revealed the core genome and species-specific genes of cutaneous propionibacteria (Figure 1; Additional file 2). 1380 proteins are common to all three species (Figure 1b). KEGG analysis showed that this core genome encodes main metabolic pathways, including the propionate formation pathway, the respiratory chain and the fatty acid metabolism (data not shown). *P. acnes* proteins encoded by the core genome show in average 89% and 73% identity to homologs of *P. avidum* and *P. granulosum*, respectively. This is in agreement with phylogenetic analyses based on 16S rRNA gene sequences, showing that *P. acnes* and *P. avidum* are closely related, whereas *P. granulosum* is more distant (data not shown).

Larger and smaller species-specific genomic islands were identified (Figure 1a; Additional files 2 and 3). Most of these are associated with a significant divergence from the main G + C content, which could indicate horizontal gene transfer (HGT) events. For example, the two genomes of *P. acnes* contain larger regions (>10 kb) not present in *P. avidum* and *P. granulosum*; these encode among others non-ribosomal peptide synthetases (PPA1277-PPA1307), and harbor genes for nitrate reductase and anaerobic dimethyl sulfoxide reductase (PPA0497-PPA0520) (Additional file 2a). Other interesting results of comparative genome analyses are reported in the next sections.

### Absence of host-interacting, putative virulence factors in the genomes of *P. avidum* and *P. granulosum*

**Figure 2:**
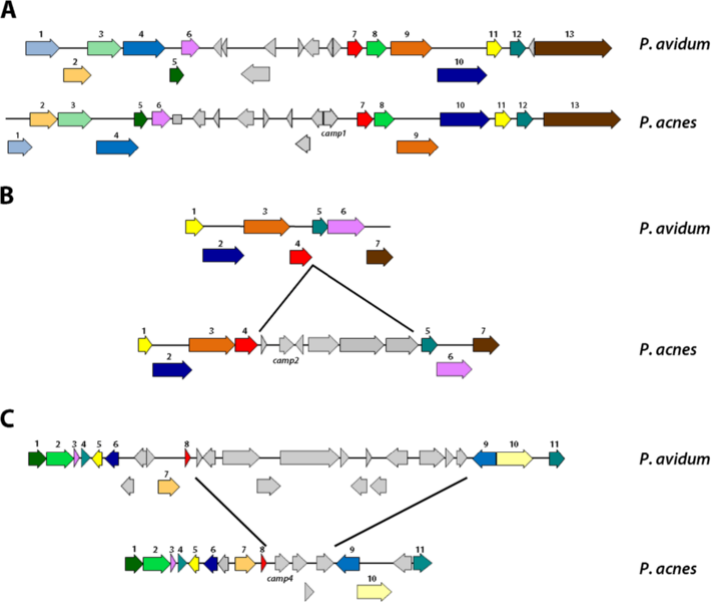
**CAMP factor encoding loci of the *P. acnes *genome are absent from the *P. avidum *genome. A)** CAMP factor 1 (PPA1340) in the *P. acnes* KPA genome is encoding within a gene cluster, that is replaced by a different cluster in *P. avidum*. Adjacent genes to this gene cluster are conserved in both genomes. Same color and number depicts homologs. **B)** CAMP factor 2 (PPA0687) of *P. acnes* is clustered with five other genes, encoding sialidases and a sialic acid transporter. This gene cluster is absent in the genome of *P. avidum*. **C)** CAMP factor 4 (PPA1231) and three adjacent genes of *P. acnes* KPA are replaced in the genome of *P. avidum* by a larger gene cluster. *P. avidum* ATCC25577 was used for this illustration; the genome of *P. avidum* TM16 is identical in the depicted genomic regions.

Genome sequencing of *P. acnes* revealed the existence of five Christie-Atkins-Munch-Petersen (CAMP) factors [16,17]. At least three of these, CAMP factors 1, 2 and 4, are produced as either secreted or surface-exposed proteins [17,23]. It has been shown that at least CAMP factor 2 has properties of a co-hemolysin and exotoxin [24,25]. Genome analysis revealed now that the genes *camp1, camp2* and *camp4* are absent from the genomes of *P. avidum* and *P. granulosum*. The latter genome carries only one CAMP factor gene, designated here CAMP factor 6 (H640_02108 in strain TM11; H641_03053 in strain DSM20700), since it has no strong similarity to one of the five CAMP factors of *P. acnes*. *P. avidum* contains two CAMP factor genes with high similarities to CAMP factors 3 and 5, respectively (HMPREF9153_0708 and HMPREF9153_1759 in strain ATCC25577). Interestingly, when comparing the genomic regions containing the *camp1, camp2* and *camp4* genes in *P. acnes* with the genome of *P. avidum*, we noticed that the CAMP factor genes are encoded on genomic island-like regions (Figure 2). For example, the *camp2* gene is inserted as part of a six-gene cluster into the backbone genome. This cluster also contains two genes encoding sialidases (PPA0684, PPA0685) and a sialic acid transporter (PPA0686). The *camp1* containing region is replaced in *P. avidum* with a region of eight genes encoding a putative arsenate reductase and transposases, underlining the mobile nature of this genomic region. The *camp4* region is replaced in *P. avidum* with an island-like region of 13 genes, encoding among others a type I restriction-modification system.

Besides the *camp* genes also other predicted host-interacting factor-encoding genes of *P. acnes* are absent from the genomes of *P. avidum* and *P. granulosum*, including a hyaluronate lyase (PPA0380), the two dermatan-sulphate adhesins DsA1 and DsA2 [18,19], and other proline-threonine repeat-motif proteins (PPA1880, PPA1715) (Additional file 4). Furthermore, the characterized sialidase (PPA1560) that has been shown to have a role in *P. acnes* adhesion and cytotoxicity [20], is truncated in *P. avidum* ATCC25577 and absent from *P. avidum* 44067 and *P. granulosum* DSM20700 and TM11. We tested the sequenced strains for hyaluronidase and neuraminidase activities: only the *P. acnes* strains were positive (data not shown), thus confirming findings from genome analyses.

### *P. avidum* produces an exopolysaccharide-like structure

**Figure 3:**
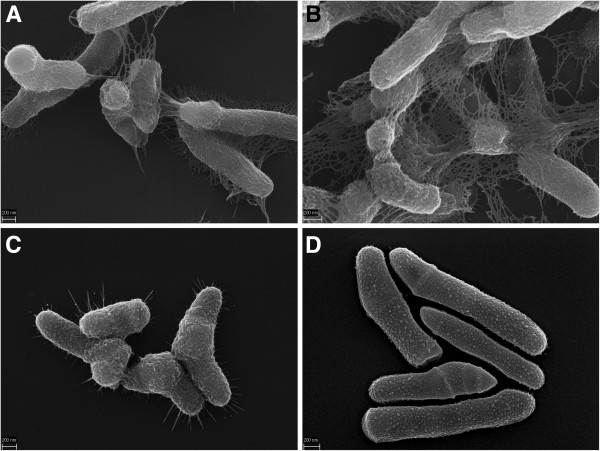
**Electron microscopic images of three propionibacteria.** Shown are representative images of **A)***P. avidum* ATCC25577; **B)***P. avidum* TM16; **C)***P. granulosum* DSM20700, and **D)***P. acnes* KPA. *P. avidum* cells are surrounded by a meshwork structure. *P. granulosum* cells have appendices similar to fimbriae or pili.

**Figure 4:**
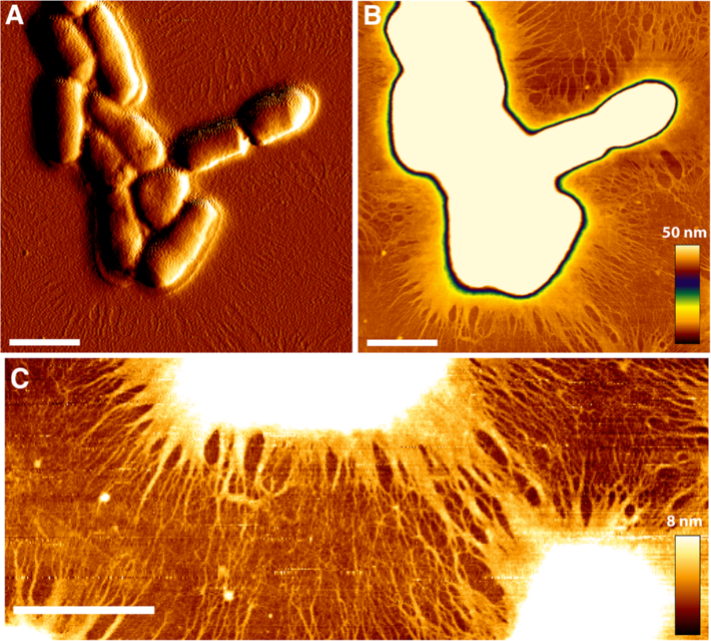
**Atomic force microscopy of *P. avidum *ATCC25577. A)** Amplitude image of the overall morphology of the cells. Extracellular structures are clearly visible, extending out from the cell surface. **B)** and **C)** Height images showing extracellular structures collapsed onto the glass coverslip. The fiber-like structures measure 1-3 nm in diameter. X-Y scalebar = 1 μm, z scale is indicated by color.

The search for species-specific genomic regions identified a large genomic island, present in all *P. avidum* genomes (HMPREF9153_1223 to HMPREF9153_1257 in strain ATCC25577; PALO_09550 to PALO_09690 in strain 44067), that is absent from *P. acnes* and *P. granulosum* (Additional file 2b). This region harbors 35 genes (in strain ATCC25577), 19 of them encode glycosyl transferases and enzymes involved in mono- or polysaccharide modification. We noticed a similarity of this cluster to a 20-gene cluster found in the genome of *Rothia mucilaginosa*, a Gram-positive, coagulase-negative coccus that is part of the commensal flora of the oral cavity and the upper respiratory tract in humans (Additional file 5a). *R. mucilaginosa* produces a thick exopolysaccharide (EPS)-like structure [26]; thus, we applied electron microscopy to examine the cell morphology of *P. avidum*. We observed a meshwork structure surrounding cells of *P. avidum* that was absent from *P. acnes* and *P. granulosum* (Figure 3), and that is very similar to the meshwork structures surrounding *R. mucilaginosa.* Atomic force microscopy further confirmed the presence of an extracellular structure of *P. avidum* extending several μm from the cell surface (Figure 4). The AFM imaging excludes the possibility of artifacts caused by the vacuum environment of the electron microscope, but the structure did collapse on to the cell surface and underlying glass substrate upon gentle air drying of the sample. It is probably a highly hydrated and amorphous material when in aqueous solution, as it appears more amorphous rather than filamentous in structure at the cell surface where some level of hydration remains. To investigate the composition of this structure, we used specific fluorescent stains for DNA and for carbohydrates (Additional file 5b), which revealed that the structure is made of polysaccharides containing β-1,4 or β-1,3 bonds.

### *P. granulosum* possesses pili-like appendices

**Figure 5:**
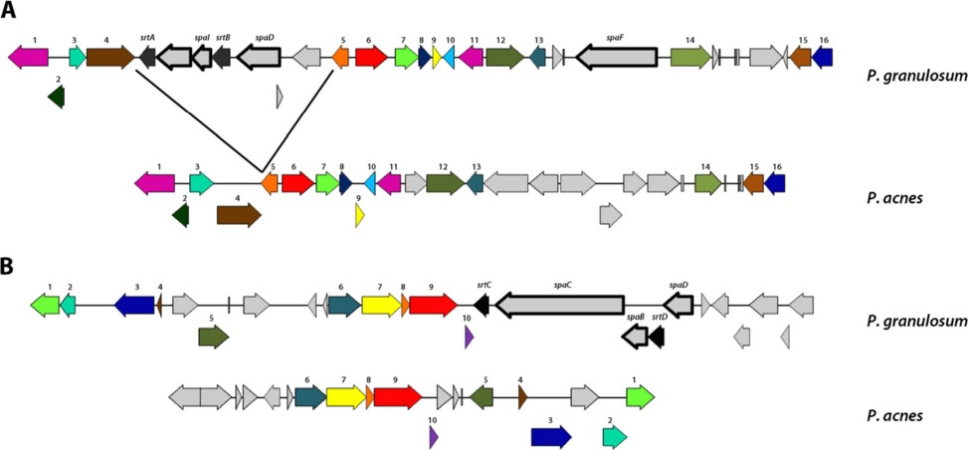
**Fimbriae/Pili-encoding loci in the genome of *P. granulosum*. A and B)** Two loci in the *P. granulosum* genome encode sortases (designated here SrtA-D). These genes are clustered with genes encoding proteins similar to fimbriae/pilin subunits of *Corynebacterium* sp. (designated here SpaB, SpaC, SpaD, SpaF and SpaI, based on their sequence similarity to pilin subunits of *Corynebacterium diphtheriae*). These proteins carry a C-terminal LPXTG motif (boxed genes). The two genetic loci are absent from the genomes of *P. acnes* KPA and 266 and *P. avidum* ATCC25577 and 44067. *P. granulosum* TM11 and *P. acnes* KPA were used for this illustration; the genomes of *P. granulosum* DSM20700 and *P. acnes* 266 are highly similar in the depicted genomic regions. Same colors and numbers depict homologies between CDS of different species.

Electron microscopy further revealed pili- or fimbriae-like appendices on the cell surface of *P. granulosum* (Figure 3C). Such structures were not detected on the surface of *P. acnes* and *P. avidum*. Pili or fimbriae are found in several Gram-positive bacteria, including some species of the related genus *Corynebacterium*[27]. In *Corynebacterium* sp. pilin subunits are encoded in several gene clusters that also contain genes for pilin-specific sortases. In a search for such gene clusters in the genome of *P. granulosum* we found two gene clusters that encode multiple sortases (designated SrtA-D; H641_03530, H641_03545, H641_05823, H641_05838 in strain DSM20700) and homologs of several pilin subunits of *Corynebacterium* sp., namely the major pilin SpaD and minor pillins SpaB, SpaC, SpaF and SpaI (Figure 5). These two clusters are absent from the genomes of *P. acnes* and *P. avidum*. The Spa-like proteins possess a typical LPXTG sorting motif for anchoring at the C-terminus, and a N-terminal signal peptide. In order to investigate which proteins are involved in pilus formation in *P. granulosum* we identified surface-attached proteins via a surfome approach (see below). Two pilin subunits were identified in strain DSM20700, namely homologs of SpaD (H641_05818) and SpaB (H641_03535) that are 33% and 35% identity to SpaD and SpaB of *Corynebacterium ulcerans* and *Corynebacterium resistens*, respectively (Table 1). The SpaD homolog could represent the major subunit of the pilus, and the SpaB homolog could be a minor pilin, in analogy to the SpaD-type pilus of *Corynebacterium diphtheria*[27].

**Table 1:** **Selected surface-associated factors of *P. avidum, P. granulosum *and *P. acnes *identified from surface proteome analysis**

**Locus tag**	**Size (kDa)**	**Function**	**Score**	**Matches**	***P. acnes *homolog**
***P. avidum *ATCC25577**					
HMPREF9153_0971	53.0	Rare lipoprotein A (RlpA family)	747	18	PPA2271 (76%)
HMPREF9153_0397	33.6	Glutamine/glutamate ABC superfamily ATP binding cassette Transporter	601	15	no
HMPREF9153_1303	60.9	Peptidase (SlpE)	349	10	PPA0247 (78%)
HMPREF9153_0820	87.7	Penicillin-binding protein PonA	321	4	PPA2149 (89%)
HMPREF9153_0967	26.2	Hypothetical protein	292	6	no
***P. granulosum *DSM20700**					
H641_02938	63.1	Rare lipoprotein A (RlpA family)	1290	24	PPA2271 (39%)
H641_04819	36.9	Hypothetical protein	1159	19	no
H641_08540	56.6	Conserved hypothetical protein	1077	13	no
H641_04393	61.1	Hypothetical protein	921	15	PPA0444 (72%)
H641_03802	41.2	Rare lipoprotein A (RlpA family)	803	11	PPA2239 (64%)
H641_06353	84.5	Phosphoesterase	540	9	PPA1745 (66%)
H641_03053	29.4	CAMP factor 6	533	8	PPA0687 (41%)
H641_07095	41.2	Putative lysophospholipase	509	7	PPA2142 (56%)
H641_05818	60.2	SpaD homolog	375	6	no
H641_03535	42.2	SpaB homolog	222	3	no
***P. acnes *KPA171202**					
PPA2175	36.5	Rare lipoprotein A (RlpA family)	2923	47	
PPA2271	52.2	Rare lipoprotein A (RlpA family)	2666	42	
PPA0644	56.6	Endoglycoceramidase	1113	13	
PPA2106	54.0	Endoglycoceramidase	1019	16	
PPA0721	40.7	NPL/P60 family secreted protein	674	12	
PPA1939	16.8	Hypothetical protein	639	12	
PPA2097	73.4	5′-nucleotidase/2′,3′-cyclic phosphodiesterase or related esterase	547	7	
PPA0687	28.6	CAMP factor 2	394	4	
PPA2105	35.9	Triacylglycerol lipase precursor	353	4	
PPA2239	41.0	Lipoprotein A-like protein	342	5	
PPA1340	30.3	CAMP factor 1	230	3	

Listed are all surface-exposed proteins that possess a typical N-terminal signal peptide. See Additional file 7 for a complete list.

### Secreted and surface-associated proteins of *P. avidum, P. granulosum* and *P. acnes*

We determined main secreted and surface-attached proteins of the three cutaneous propionibacteria, since such proteins could mediate the contact with human tissue sites, and could reveal host-interacting strategies. For determining secreted proteins, we collected culture supernatants, precipitated the secreted proteins and identified prominent bands on a 1D-SDS-PAGE gel by mass spectrometry (Additional file 6). For *P. avidum* ATCC25577 the main secreted protein, under the applied growth conditions, is a triacylglycerol lipase that is 48% similar to GehA (PPA2105), a characterized lipase of *P. acnes*. Furthermore, a homolog of CAMP factor 3 and several proteins of unknown function could be detected in the supernatant of *P. avidum* ATCC25577 (Additional file 7). All identified eight secreted proteins of *P. avidum* have a homolog in *P. acnes;* five of eight homologs have also been detected in *P. acnes* supernatants [23]. *P. granulosum* DSM20700 abundantly secretes two proteins, with peaks in the beginning of the stationary phase: two predicted lysophospholipases that are 53% and 56% similar to a putative lysophospholipase (PPA2142) of *P. acnes* (Additional files 6 and 7). Homologs of all secreted proteins in *P. granulosum* have been detected in culture supernatants of *P. acnes*[23]. Homologs of one protein (PPA0532) are secreted by all cutaneous propionibacteria; the function of PPA0532 is unknown. Our previous study has shown that *P. acnes*, regardless of the specific subtype, abundantly secretes CAMP factor 2, a putative lysozyme (PPA1662), an endoglycoceramindase (PPA0644), and a protein of unknown function (PPA1939) [23]. Homologs of none of these proteins were detected in culture supernatants of *P. avidum* ATCC25577 or *P. granulosum* DSM20700; the corresponding genes are absent from their genomes (Figure 2; Additional file 2a). This indicates that *P. acnes* secretes a unique set of factors.

**Figure 6:**
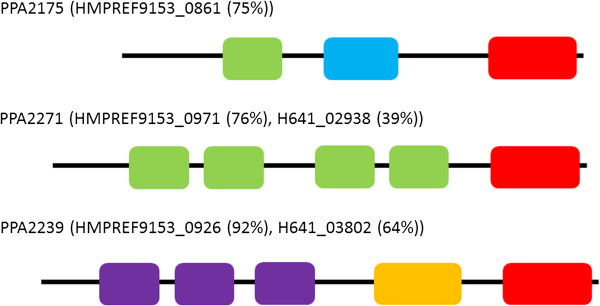
**Domain architecture of surface-attached lipoproteins of propionibacteria.** Propionibacteria produce lipoproteins with a RlpA domain that are attached to the cell surface. *P. acnes* KPA has three orthologs (PPA2172, PPA2271, PPA2239); these proteins are abundantly detected on the surface of *P. acnes* (PPA2175, PPA2271) or secreted (PPA2239). In brackets: orthologs in *P. avidum* ATCC25577 (HMPREF9153_X) and *P. granulosum* DSM20700 (H641_X). Legend: red, RlpA domain; green, SH3 domain; blue, peptidoglycan-binding domain; lila, DUF348 domain; orange, G5 domain.

The surface-exposed proteins were determined by a surfome-approach that is based on trypsin cleavage of surface-exposed protein moieties [28]. In all three *Propionibacterium* species the most abundantly detected surface-attached proteins were RlpA-domain containing lipoproteins (Table 1; Additional file 7). Two such proteins (PPA2175 and PPA2271) were detected on the surface of *P. acnes*. PPA2175 is exclusively produced by *P. acnes*; it contains a bacterial SH3 and a peptidoglycan-binding domain (Figure 6). The corresponding gene is absent from the genomes of *P. avidum* and *P. granulosum*. Homologs of the other lipoprotein, PPA2271, were also abundantly detected on *P. granulosum* DSM20700 (H641_02938) and *P. avidum* ATCC25577 (HMPREF9153_0971). Additional species-specific surface-attached factors were identified, e.g. CAMP factor 6 of *P. granulosum*. In *P. acnes*, CAMP factors 1 and 2 were detected as well as two endoglycoceramidases and two GroEL chaperonins (Additional file 7). The duplication of surface-exposed factors points to some redundancy, and could indicate that the duplicated factors are of high importance for *P. acnes*.

## Discussion

Here, we analyzed and compared the genomes of the three propionibacterial species known to colonize the human skin. Species-specific gene clusters were identified in each genome that encode traits for colonization and host-interaction. Applying high-resolution microscopy and proteomic approaches we could verify the production of these surface-associated functions.

*P. avidum* was found to be surrounded by an EPS-like meshwork. A gene cluster that encodes proteins involved in the biosynthesis and modification of EPS was identified. The cluster encodes several homologs for enzymes involved in LPS and EPS biosynthesis (RfbA, RfbB, RfbD, RfaG, ExoU, NeuA, NeuB) as well as a number of glycosyl transferases with unknown specificities. RfbA, B, D are found in *Lactococcus lactis*, and required for dTDP-rhamnose biosynthesis, which is an important precursor of rhamnose-containing exopolysaccharides [29]. The genes *neuA* and *neuB* are found in the LPS biosynthesis gene clusters of several Gram-negative species. NeuA and NeuB have been shown to be important in polysialic acid capsule biosynthesis [30]. The *P. avidum* EPS gene cluster lacks a gene for a flippase, indicating that the EPS structure is formed on the outside of the cell. Interestingly, the cluster also contains genes involved in trehalose biosynthesis (TreY-TreZ pathway). The disaccharide trehalose can protect cells from environmental stresses such as low water availability [31]. 20 of the 35 genes have a homolog in *R. mucilaginosa* that produces a mucilaginous capsular material [26]. Like *P. avidum, R*. *muciloginosa* is occasionally isolated from disease sites, thus regarded as an opportunistic pathogen, for instance involved in prosthetic device infections. Well-studied EPS in other bacteria, such as in *Pseudomonas aeruginosa*, have several roles in pathogenicity; EPS contributes to biofilm formation, adherence to surfaces and host cells, evasion of phagocytosis, and elicitation of immune response [32-34]. We hypothesize that the EPS structure of *P. avidum* could have a role in biofilm formation, and thereby contribute to its pathogenicity by leading to persistent infections that cannot be cleared by the immune system. This might explain why *P. avidum* is in particular recognized in abscess formation after surgical intervention [11-13,22]. It should be noted that several studies reported the presence of a cell wall-associated polysaccharide of *P. acnes* that can partially be extracted by phenol extraction [35-37]. Such a cell wall polysaccharide was further described as a lipidated macroamphiphile; this lipoglycan cell envelope component of *P. acnes* was found to have a lipid anchor and a polysaccharide moiety containing mannose, glucose and galactose, and probably diaminohexuronic acid [38]. We strongly suspect that the lipoglycan of *P. acnes* is distinct from the EPS of *P. avidum*. We tested different strains of *P. acnes* grown under different conditions and could not detect any EPS-like structure by EM analyses (data not shown). To our knowledge no study so far could visualize an EPS-like meshwork on *P. acnes* cells. Moreover, the identified putative EPS biosynthesis genes of *P. avidum* are absent from the genomes of *P. acnes* (and *P. granulosum*).

We found that *P. granulosum* possesses pili/fimbriae-like appendices and pilin subunits were identified among cell surface-exposed proteins of *P. granulosum*. We determined two gene clusters encoding pilin subunits in direct vicinity to genes encoding sortases. Such clustering of genes for sortase and pilin subunits has been reported for a number of Gram-positive bacteria, including related actinobacteria such as *Corynebacterium diphtheriae* that produces three distinct pilus structures, SpaA-, SpaD- and SpaH-type pili [27]. In corynebacteria pilins are covalently polymerized and the formed pilus is anchored to the bacterial cell wall; these steps are catalyzed by pilin-specific and housekeeping sortases, respectively. It has been shown that minor pilins (SpaB/SpaC) represent the major adhesins of corynebacteria [27,39]. Thus, we hypothesize that pili of *P. granulosum* could have a role in adhesion to human skin tissue and colonization. They might also have a role in forming a multispecies biofilm, since *P. granulosum* and *P. acnes* are often detected together within sebaceous follicles. The anchorage of the base of the pilus to the cell wall is usually mediated by a housekeeping sortase [27]. A likely candidate for this housekeeping sortase was identified among the surface-associated proteins: H641_09423 (strain DSM20700), a protein with a sortase E domain. A homolog exists in *P. acnes* KPA (PPA0777) and in *P. avidum* ATCC25577 (HMPREF9153_2132). These sortases likely catalyze the anchoring of other LPXTG-motif proteins of the three propionibacterial species to their cell walls. A genome search revealed that *P. avidum* contains 12, *P. acnes* 15, and *P. granulosum* 18 proteins (including 7 putative pilin subunits) with a C-terminal LPXTG motif. Most of these LPXTG-motif proteins have no or little similarity to known proteins, exceptions are proteins with nucleotidase or phosphoesterase domains. Only few of these LPXTG-motif proteins have been identified in the surfome. That could either indicate that they were not or weakly expressed under the applied growth conditions (liquid culture, complex broth), or were not accessible for trypsin cleavage.

Analysis of the surfome data of *P. granulosum* further revealed the presence of several cytosolic proteins, including ribosomal proteins and those involved in core metabolic functions (methylmalonyl-CoA:pyruvate transcarboxylase 12S subunit; two methylmalonyl-CoA mutases; fumarate hydratase class II; succinyl-CoA ligase). That indicates that *P. granulosum* seems to be more sensitive to trypsin treatment or lyse earlier than *P. avidum* and *P. acnes*.

*P. acnes* has, unlike *P. granulosum* and *P. avidum*, no obvious surface appendices. However, *P. acnes* is by far the most prevalent bacterium in sebaceous follicles of the face and back [2,7]. Thus, this species must have evolved a different strategy to adhere to and colonize human tissues. Surface proteins could act as powerful adhesins. Indeed, the dermatan-sulphate adhesins DsA1 and DsA2 have been identified and partially characterized in *P. acnes*[18,19]. These were not found in the surfome of the strain KPA, most likely because the respective genes are phase variable, but DsA1 and DsA2 are present on the surface of the type Ia strain 266 (data not shown). In addition, the surfome data revealed an abundance of lipoproteins with RlpA (rare lipoprotein A) domains on the surface of *P. acnes*. The bacterium specifically produces PPA2175 on the surface; it contains a SH3 and a peptidoglycan-binding domain (Figure 6). Bacterial lipoproteins have diverse functions; they play roles in a wide range of physiological processes. They can also function as ligands of the innate immunity host cell receptor Toll-like receptor 2 (TLR2), thus triggering an innate immune reaction [40]. It has been reported that TLR2 was sufficient for NF-kappaB activation in response to *P. acnes* and activation of TLR2 resulted in an inflammatory cytokine response, which is thought to be of crucial importance in acne vulgaris [41,42]. The TLR2 ligand of *P. acnes* is so far unknown. We speculate that one or all of the surface-exposed RlpA-domain lipoproteins of *P. acnes* are TLR2 ligands. These lipoproteins are abundantly produced on the surface of *P. acnes* and they are not covered or protected from host cell contact by other surface structures, such as EPS or pili in *P. avidum* and *P. granulosum*, respectively. It will be interesting to investigate if *P. avidum* and *P. granulosum* are also able to trigger TLR2 responses, or if this is specific to *P. acnes*.

The colonization of human skin by *P. acnes* can be achieved by other strategies, such as factors that allow successful competition with other bacteria, including *P. avidum* and *P. granulosum*. Successful competition might include the efficient acquisition of nutrients from host components. In this respect, only *P. acnes* expressed surface-attached endoglycoceramidases, which might hydrolyze gangliosides on host cell membranes [43]. Another specific feature of *P. acnes* is the presence and the production of surface-exposed CAMP factors 1 and 2. It has been shown that CAMP factor 2 has properties of a co-hemolysin [17,24,25,44]. Moreover, inhibition of CAMP2 by neutralizing antibodies efficiently attenuated *P. acnes*-induced inflammation in the mouse ear model [45], suggesting that CAMP2, and probably the other ones as well, are virulence factors of *P. acnes*. The corresponding *camp1* and *camp2* genes are absent in the genomes of *P. granulosum* and *P.avidum*. CAMP factors have been partially characterized in streptococcal species as co-hemolsyins and pore-forming toxins [46]. They are involved in the CAMP reaction, the lysis of sheep erythrocytes by the synergistic action of the sphingomyelinase C from *S. aureus* and CAMP factor from Group B *Streptococcus* strains [47]. The sphingomyelinase initially hydrolyzes sphingomyelin to ceramide (and phosphocholine) on the erythrocyte membrane, which renders the erythrocytes susceptible to the lytic activity of CAMP factor. It was recently shown that CAMP factor 2 of *P. acnes* can act as an exotoxin, exhibiting cytotoxic activity on host cells [25]. The study of Nakatsuji et al. further suggests that CAMP factor 2 acts together with host acid sphingomyelinase to amplify bacterial virulence, thus supporting the degradation and invasion of host cells. The gene for CAMP factor 2 is located within a small gene cluster that also contains genes encoding sialidases and a sialic acid transporter. This cluster seems to be inserted into the *P. acnes* genome (Figure 2). It is tempting to suggest a functional connection of these factors as host-interacting and/or virulence traits. One possible scenario is that sialidases act directly on host cell membrane exposed gangliosides, thus releasing terminal sialic acid residues that are taken up by the sialic acid transporter and used as energy source. The remaining ceramide moiety could be a binding site for CAMP factor 2, in analogy to the CAMP reaction. Another component in this scenario could be surface-associated endoglycoceramidases of *P. acnes* that is predicted to hydrolyze gangliosides on host cell membranes into ceramides and oligosaccharides.

Important questions remain to be answered, in particular regarding the host tissue and host cell interactions of these three species. Although all three species are colonizing human skin, it is not known if these species actually compete at those sites or have adapted to occupy unique niches through species-specific host interactions. The different surface properties of the three species suggest that they have different colonization strategies that could be host cell or tissue-specific at skin and non-skin sites.

## Conclusions

Taken together, comparative genome analysis showed that CAMP factor 1 and 2 and other host-interacting traits (e.g. DsA1, DsA2, hyaluronate lyase, endoglycoceramidase, sialidase, linoleic acid isomerase) are encoded on smaller genomic regions that are either inserted into the genome of *P. acnes* or deleted from the genomes of *P. avidum* and *P. granulosum*. That might explain the greater versatility of *P. acnes* to interact with the human host. *P. avidum* and to a minor extent *P. granulosum* have their own species-specific genomic regions, that are absent from *P. acnes*. Among these are the EPS cluster of *P. avidum* and the pili/fimbriae gene clusters of *P. granulosum*. Thus, human-associated propionibacteria have evolved different host-interacting strategies which are likely linked to different disease-causing potentials of these three species.

## Methods

### Bacteria strains and culture

*P. avidum* strain ATCC25577 and *P. granulosum* strain DSM20700 were obtained from DSMZ (German Collection of Microorganisms and Cell Cultures). *P. acnes* KPA171202 (KPA, type I-2) and *P. acnes* 266 (type IA) were previously isolated [16,21]. *P. avidum* TM16 and *P. granulosum* TM11 were isolated from radical prostatectomy specimens in our previous study [10]. All strains were cultured on Reinforced Clostridial Agar (Oxoid) plates for 3 days at 37°C under anaerobic conditions. For liquid cultures, plate-grown bacteria were resuspended and washed in brain heart infusion (BHI) broth (Sigma-Aldrich); BHI broth was inoculated with *P. avidum* ATCC25577 and *P. granulosum* DSM20700 (OD_600_ 0.01) and cultures were grown to exponential (OD_600_ 0.3-0.4) and stationary phases (OD_600_ 0.9-1.2) at 37°C under anaerobic conditions using the Gas-Pak™ system (Oxoid). To ensure identical growth conditions due to the usage of the Gas-Pak system, all strains were cultured in the same GasPak container and the same BHI batch was used.

### DNA extraction and genome sequencing

Genomic DNA from all strains of *P. avidum* TM16 and *P. granulosum* DSM20700 and TM11 were extracted using the MasterPure™ Gram Positive DNA Purification Kit (Epicentre). The genomes were draft sequenced using Illumina/Solexa GAIIx machines at the Beijing Genomics Institute (BGI) (Shenzhen, China). The whole genome shotgun projects have been deposited at DDBJ/EMBL/GenBank under the accession numbers AOUA00000000 for *P. avidum* TM16, AOST00000000 for *P. granulosum* TM11 and AOSS00000000 for *P. granulosum* DSM20700. The versions described in this paper are the first versions, AOUA01000000, AOST00000000 and AOSS01000000, respectively.

### Sequence analysis

Automatic annotations were performed by PGAAP, NCBI Prokaryotic Genome Automatic Annotation Pipeline [48]. To identify homologs in different species, comparisons were done with a protein sequence-based bidirectional BLAST approach (blastP version 2.2.18). Sequence homologies were only mentioned in this study for proteins with an amino acid identity of >25% and an overlap of the query and subject sequence of >75%. Genome representations were created by DNA plotter (Sanger Institute). For nucleotide sequence comparisons the Artemis Comparison Tool (ACT) was used [49]. To identify genomic islands the Island Viewer was used, a computational tool that integrates three different genomic island prediction methods, i.e. IslandPick, IslandPath-DIMOB, and SIGI-HMM [50]. For the analysis and mapping of pathways of *P. acnes* KEGG and KEGG MAPPER were used (http://www.genome.jp/kegg/).

### Scanning electron microscopy

Bacterial cells were fixed with 2.5% glutaraldehyde, post-fixed using repeated incubations with 1% osmium tetroxide/1% tannic acid, dehydrated with a graded ethanol series, critical-point dried and coated with 2 nm platinum. After dehydration and critical-point drying, the specimens were coated with 5 nm platinum/carbon and analyzed in a Leo 1550 scanning electron microscopy.

### Atomic force microscopy, fluorescence staining and confocal microscopy

A NanoWizard II atomic force microscope (JPK Instruments, Germany) combined with an inverted optical microscope (Zeiss Axiovert 200 M, Zeiss, Germany) was used to record AFM images at 512 pixels per line, with 1 Hz scanning speed. Tapping mode in air was performed for imaging, using OMCL-AC160TS cantilevers (Olympus) with spring constant of 26 N/m. A glass coverslip was immersed in bacterial cells suspended in water, gently rinsed with water, briefly dried in air, and mounted for AFM imaging of the cells.

Staining of DNA and carbohydrates in the EPS was done by suspending bacterial cells in PBS and staining simultaneously with Calcofluor white (100 mg/l, Sigma-Aldrich) and propidium iodide (0.05 mM, Invitrogen) for 30 min. After one wash in PBS, the samples were resuspended with PBS and visualized using a confocal laser scanning microscope (LSM 700, Carl Zeiss), using 405 nm excitation for Calcofluor white, and 555 nm excitation for propidium iodide.

### Precipitation of extracellular proteins

The exponential and stationary cultures were cen-trifuged for 15 min at 6,000 × *g* and 4°C. Supernatant was filtered through a 0.22-μm-pore-size membrane filter to remove residual bacteria. Extracellular proteins were precipitated using a modified trichloroacetic acid (TCA) method [51]. In brief, the filtrate (100 ml) was mixed with 25% TCA to a final concentration of 6% and incubated overnight at -20°C. The mixture was centrifuged for 30 min (6,000 × *g* and 4°C) and the resulting pellet was resuspended in 1 ml of acetone. The mixture was centrifuged for 15 min (20,000 × g and 4°C), washed twice with acetone and the resulting pellet was air dried. The pellets were dissolved in PBS buffer and Laemmli sample before analyzed by SDS PAGE.

### Protein identification by MALDI-TOF-MS

SDS PAGE was carried out with 12% acrylamide concentration of gels. Gels were stained with Coomassie Blue. Protein bands were excised for in-gel tryptic digestion. After concentration, the peptides were loaded onto the MALDI target plate using dried droplet method and analysed by MALDI-TOF-MS on a Bruker Autoflex (Bruker Daltonik) operating in reflector mode. Peptide mass fingerprinting (PMF) and MS/MS data were searched against a manually created propionibacteria database. Proteins were identified using MASCOT 2.3 (http://www.matrixscience.com) allowing a peptide mass tolerance of 100-300 ppm and ± 0.3 Da for the fragment mass tolerance. A maximum of one missed cleavage, oxidation of methionine, N-terminal acetylation of the peptide, propionamide at cysteine residues and N-terminal pyroglutamic acid formation were considered in these searches. The identification criteria were: minimum 30% sequence coverage; or minimum 15% sequence coverage and one MS/MS confirmation; or sequence coverage below 15% and at least two MS/MS confirmations.

### Surfome analysis

The protocol for bacterial surface digestion was adapted from Doro et al. [28]. *P. avidum* strain ATCC25577, *P. acnes* KPA and *P. granulosum* strain DSM20700 strains were grown to OD_600_ 0.3 - 0.4. Bacterial cells were harvested by centrifugation at 3,500 × *g* for 10 min at 4°C and washed twice with PBS. Cells were resuspended in 800 μl of PBS containing 40% sucrose. Digestions were carried out with 10 μg of trypsin (Promega) for 30 min at 37°C. Bacterial cells were centrifuged at 3,500 × *g* for 10 min at 4°C and the supernatants were filtered through 0.22-μm pore size filters (Millipore). Protease reactions were stopped with formic acid at 0.1% final concentration. Before protein identification, PBS and sucrose were removed using ZipTip C18, 0.6 μl bed volume (Millipore). Peptides were eluted with 5 μl 60% ACN, 0.1% TFA followed by 5 μl 80% ACN, 0.1% TFA. The combined eluates were concentrated using a Microconcentrator 5301 (Eppendorf) and kept at −20°C until further analysis.

### Protein identification by UPLC/MS/MS

The samples were solubilized in 12 μl 2:98 (v/v) acetonitrile/water containing 0.1% TFA (v/v). After concentration on a Acclaim PepMap 100 trap column at a flow rate of 5 μl/min (75 μm x 2 cm, C18, 3 μm, 100 Å, Thermo Scientific) separation was performed by UHPLC (UltiMate 3000, Dionex) using a Acclaim PepMap RSLC column at a flow rate of 300 nl/min (75 μm x 150 mm, C18, 2 μm, 100 Å, Thermo Scientific). Mobile phase A was 0.1% (v/v) TFA and B was 80:20 (v/v) acetonitrile/water containing 0.08% (v/v) TFA. The elution gradients were 3-15% B for 2 min, 15-60% B for 60 min, 60-98% B for 4 min, 98% B for 2 min and 98-3% B for 3 min. 312 fractions per sample were spotted onto a MALDI template using a Probot microfraction collector (Dionex). Spotting frequency was 10 seconds and α-cyano-4-hydroxycinnamic acid (0,5% in 70:30 (v/v) acetonitrile/water containing 0.1% (v/v) TFA) was added at a flow rate of 1 μl/min. Mass spectra were acquired with a 4700 Proteomics Analyzer (Applied Biosystems) MALDI-TOF/TOF instrument. The MS mass range was 800-4000 Da. MS/MS precursor selection was performed automatically; using the 4000 Series Explorer Software 3.6 and a maximum of 7 MS/MS measurements per spot were allowed. MS/MS data were searched against a manually created propionibacteria database using MASCOT 2.3 (Matrix Science) allowing a peptide mass tolerance of 100-150 ppm and 0.3 Da for the fragment mass tolerance. Enzyme specificity was set to none. N-acetyl (Protein), oxidation (M), pyro-glu (N-term Q) were considered in these searches and standard scoring and ions score cut-off 30 were used for data evaluation. The criterion for the identification of a protein was a minimum number of 3 peptides fulfilling the Mascot homology criteria.

### Availability of supporting data

The Whole Genome Shotgun projects have been deposited at DDBJ/EMBL/GenBank under the accession AOST00000000 (http://www.ncbi.nlm.nih.gov/bioproject/PRJNA189037), AOSS00000000 (http://www.ncbi.nlm.nih.gov/bioproject/PRJNA189038) and AOUA00000000 (http://www.ncbi.nlm.nih.gov/bioproject/PRJNA189036). Other supporting data are included as Additional files 1, 2, 3, 4, 5, 6 and 7.

## Abbreviations

CAMP: Christie-Atkins-Munch-Petersen; EPS: Exopolysaccharide.

## Competing interests

The authors declare that they have no competing interests.

## Authors’ contributions

TNM and HB conceptualized and designed the study and analyzed data. MS carried out mass spectrometry experiments and analyzed data. EB and HB analyzed the genomes. GZ and RM performed atomic force microscopy. KSS and TFM provided material and advice. VB performed electron microscopy. TNM and HB wrote the manuscript and all authors reviewed and edited the manuscript. All authors read and approved the final manuscript.

## Supplementary Material

Additional file 1 Genome features of the propionibacterial species *P. avidum, P. granulosum* and *P. acnes.* Click here for file

Additional file 2 A) Bidirectional Blast of all CDS of *P. acnes* KPA against other genomes of *P. acnes, P. granulosum* and *P. avidum.* B) Bidirectional Blast of all CDS of *P. avidum* 44067 against other genomes of *P. avidum, P. acnes* and *P. granulosum.* C) Bidirectional Blast of all CDS of *P. granulosum* TM11 against other genomes of *P. granulosum, P. acnes* and *P. avidum.* The color code represents the Blast e-values: White: >e-20; Light yellow: <e-20 and > e-50; Gold: <e-50 and > e-90; Light orange: <e-90 and > e-100; Orange: <e-100 and > e-120; Red: <e-120. Click here for file

Additional file 3 Comparative genome analysis of three cutaneous propionibacteria. A) Genome comparison with *P. granulosum* TM11 as the reference genome. B) Genome comparison with *P. acnes* KPA as the reference genome. Color code: CDS of *P. granulosum* TM11, marine; *P. granulosum* DSM20700, green; *P. avidum* ATCC25577, red; *P. avidum* TM16, orange; *P. acnes* KPA, blue; *P. acnes* 266, light blue. The inner ring (in purple and olive) represents the G + C content distribution of the reference genome (window size 10000 bp, step size 200 bp). The most inner circle depicts predicted islands (in red) acquired by horizontal gene transfer (predictions from IslandViewer; results from two different algorithms are included: orange, Sigi-HMM; blue, IslandPath-DIMOB). *P. granulosum* and *P. acnes* harbors 10 and 5 genomic regions, respectively, that are predicted to be horizontally acquired. Click here for file

Additional file 4 Genes encoding host-interacting proteins of *P. acnes* are absent from the genome of *P. avidum*. Shown are four examples of genomic regions encoding putative host-interacting proteins of *P. acnes* that differ or are deleted in the genome of *P. avidum* ATCC25577. A) This *P. acnes*-specific region encodes a hyaluronidase (PPA0380) and contains 9 genes (PPA0372-PPA0382); several of them encode oxidoreductases and one encodes a glycosyl transferase. B) DsA1 (PPA2127) is a dermatan-sulphate adhesin with proline-threonine repeats [18]. The corresponding *P. acnes*-specific gene is replaced in *P. avidum* ATCC25577 by a larger island, encoding mostly proteins with unknown functions. C) DsA2 (PPA2210) is another dermatan-sulphate adhesin that is encoded in a region of 12 *P. acnes*-specific genes that includes five genes putatively involved in carnitine catabolism. D) PPA1560 encodes the characterized sialidase of *P. acnes*[19]. *P. avidum* strain ATCC25577 encodes a different sialidase (HMPREF9153_0188; 63% protein identity to PPA1560). In the genome of *P. avidum* 44067, the sialidase-encoding region is deleted (data not shown). Red bars/lines identify regions with high sequence similarity (>70%). Click here for file

Additional file 5 *P. avidum* produces an EPS structure. A) The genome of *P. avidum* harbors a gene cluster for exopolysaccharide biosynthesis (HMPREF9153_1223 to HMPREF9153_1257 in strain ATCC25577 and PALO_09550 to PALO_09690 in strain 44067). Most of the genes encode glycosyltransferases. See Additional file 2b for the functional assignment of all CDS. A similar gene cluster exists in the genome of *Rothia mucilaginosa,* a Gram-positive bacterium producing a mucilaginous capsular material. Same colors and numbers depict homologies between CDS of *P. avidum* and *R. mucilaginosa*. B) Staining experiments using calcofluor white and propidium iodide were performed, recorded by confocal microscopy (z-series, layers taken from the surface of the cells (top left) to the center of the cells (bottom right)), to confirm the existence of a polysaccharide structure surrounding *P. avidum* ATCC25577 cells. Blue, polysaccharide; red, DNA. Click here for file

Additional file 6 Secreted proteins of *P. avidum* and *P. granulosum*. A) *P. avidum* ATCC25577 and B) *P. granulosum* DSM20700 were grown in BHI medium to exponential (E) and early stationary (S) phase. Secreted proteins were precipitated from culture supernatants and separated on a SDS-PAGE gel (12%). Abundant bands (numbered) were subjected to MS identification (see Additional file 7 for all identified proteins). Under the applied growth conditions, the most abundantly secreted proteins of *P. avidum* and *P. granulosum* are a triacylglycerol lipase (band 5 in section A) and two lysophospholipases (bands 1 and 2 in section B), respectively. Click here for file

Additional file 7 Identification of secreted and surface-exposed proteins of *P. avidum, P. granulosum* and *P. acnes.* Click here for file
