# Supplementary material for: Comparative genomics reveals distinct host-interacting traits of three major human-associated propionibacteria
Source: BMC Genomics. 2013 Sep 22;14:640. doi: 10.1186/1471-2164-14-640 (PMC3848858; doi:10.1186/1471-2164-14-640)
Supplement: Additional file 1 — Genome features of the propionibacterial species P. avidum, P. granulosum and P. acnes. [file 1471-2164-14-640-S1.docx]

**Additional file 1**

**Genome features of the propionibacterial species *P. avidum, P. granulosum* and *P. acnes***

| **Feature** | *P. avidum* ATCC25577 | *P. avidum*  TM16 | *P. avidum*  44067 | *P. granulosum* DSM20700 | *P. granulosum*  TM11 | *P. acnes* KPA171202 | *P. acnes* 266 |
| --- | --- | --- | --- | --- | --- | --- | --- |
| (pseudo-)chromo-some size (bp) | 2.55 | 2.56 | 2.53 | 2.13 | 2.14 | 2.56 | 2.49 |
| coverage | 29 | 34 | 375 | 45 | 92 | nd | nd |
| number of contigs | 24 | 440 | 1 | 386 | 122 | 1 | 1 |
| G+C (%) | 63.4 | 62.8 | 63.5 | 61.9 | 63.9 | 60.0 | 60.0 |
| number of CDS * | 2376 (nd) | 2491 (PGAAP) | 2242 (PGAAP) | 1962 (PGAAP) | 1803 (PGAAP) | 2297 (ERGO) | 2345 (ERGO) |
| CRISPR/Cas | I-E, I-U | I-U | I-E | I-E | I-E | none | none |
| GenBank accession # | AGBA00000000 | AOUA00000000 | CP005287 | AOSS00000000 | AOST00000000 | AE017283.1 | CP002409.1 |

* in brackets: annotation tool; PGAAP, NCBI Prokaryotic Genome Automatic Annotation Pipeline; ERGO, ERGO™ genome analysis and discovery system (http://www.integratedgenomics.com). Note that differences in contig numbers affect gene prediction accuracy. The number of CDS is higher for genomes split into many contigs, due to incomplete genes at the contig boundaries.
