# Supplementary figures and images for: Comparative genomics reveals distinct host-interacting traits of three major human-associated propionibacteria
Source: BMC Genomics. 2013 Sep 22;14:640. doi: 10.1186/1471-2164-14-640 (PMC3848858; doi:10.1186/1471-2164-14-640)

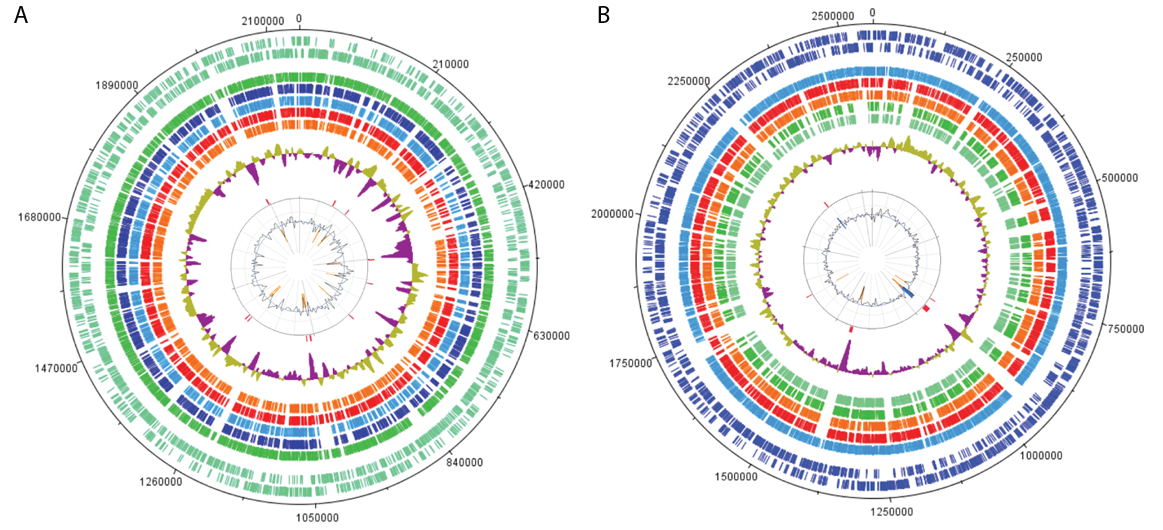

Supplement: Additional file 3 — Comparative genome analysis of three cutaneous propionibacteria. A) Genome comparison with P. granulosum TM11 as the reference genome. B) Genome comparison with P. acnes KPA as the reference genome. Color code: CDS of P. granulosum TM11, marine; P. granulosum DSM20700, green; P. avidum ATCC25577, red; P. avidum TM16, orange; P. acnes KPA, blue; P. acnes 266, light blue. The inner ring (in purple and olive) represents the G + C content distribution of the reference genome (window size 10000 bp, step size 200 bp). The most inner circle depicts predicted islands (in red) acquired by horizontal gene transfer (predictions from IslandViewer; results from two different algorithms are included: orange, Sigi-HMM; blue, IslandPath-DIMOB). P. granulosum and P. acnes harbors 10 and 5 genomic regions, respectively, that are predicted to be horizontally acquired. [file 1471-2164-14-640-S3.tiff]

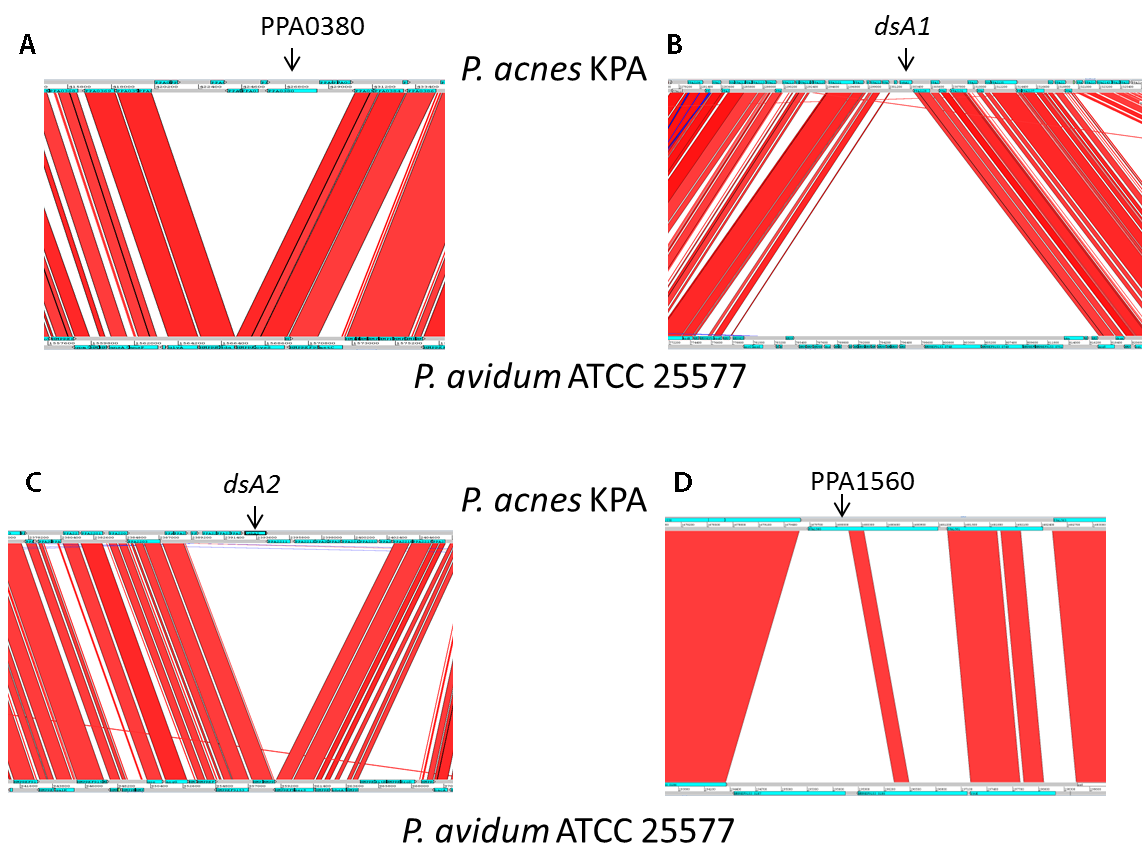

Supplement: Additional file 4 — Genes encoding host-interacting proteins of P. acnes are absent from the genome of P. avidum. Shown are four examples of genomic regions encoding putative host-interacting proteins of P. acnes that differ or are deleted in the genome of P. avidum ATCC25577. A) This P. acnes-specific region encodes a hyaluronidase (PPA0380) and contains 9 genes (PPA0372-PPA0382); several of them encode oxidoreductases and one encodes a glycosyl transferase. B) DsA1 (PPA2127) is a dermatan-sulphate adhesin with proline-threonine repeats [18]. The corresponding P. acnes-specific gene is replaced in P. avidum ATCC25577 by a larger island, encoding mostly proteins with unknown functions. C) DsA2 (PPA2210) is another dermatan-sulphate adhesin that is encoded in a region of 12 P. acnes-specific genes that includes five genes putatively involved in carnitine catabolism. D) PPA1560 encodes the characterized sialidase of P. acnes[19]. P. avidum strain ATCC25577 encodes a different sialidase (HMPREF9153_0188; 63% protein identity to PPA1560). In the genome of P. avidum 44067, the sialidase-encoding region is deleted (data not shown). Red bars/lines identify regions with high sequence similarity (>70%). [file 1471-2164-14-640-S4.tiff]

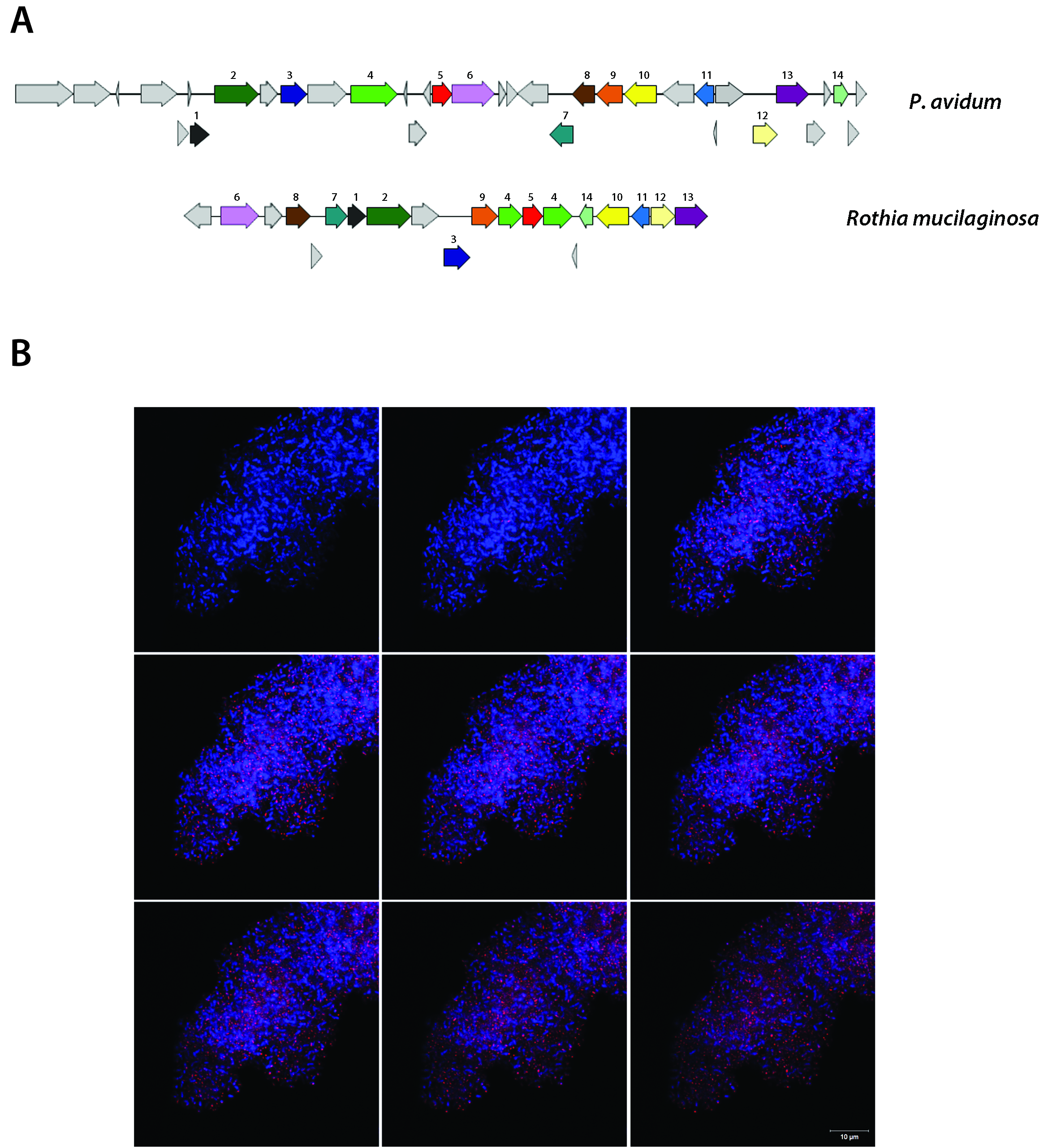

Supplement: Additional file 5 — P. avidum produces an EPS structure. A) The genome of P. avidum harbors a gene cluster for exopolysaccharide biosynthesis (HMPREF9153_1223 to HMPREF9153_1257 in strain ATCC25577 and PALO_09550 to PALO_09690 in strain 44067). Most of the genes encode glycosyltransferases. See Additional file 2b for the functional assignment of all CDS. A similar gene cluster exists in the genome of Rothia mucilaginosa, a Gram-positive bacterium producing a mucilaginous capsular material. Same colors and numbers depict homologies between CDS of P. avidum and R. mucilaginosa. B) Staining experiments using calcofluor white and propidium iodide were performed, recorded by confocal microscopy (z-series, layers taken from the surface of the cells (top left) to the center of the cells (bottom right)), to confirm the existence of a polysaccharide structure surrounding P. avidum ATCC25577 cells. Blue, polysaccharide; red, DNA. [file 1471-2164-14-640-S5.tiff]

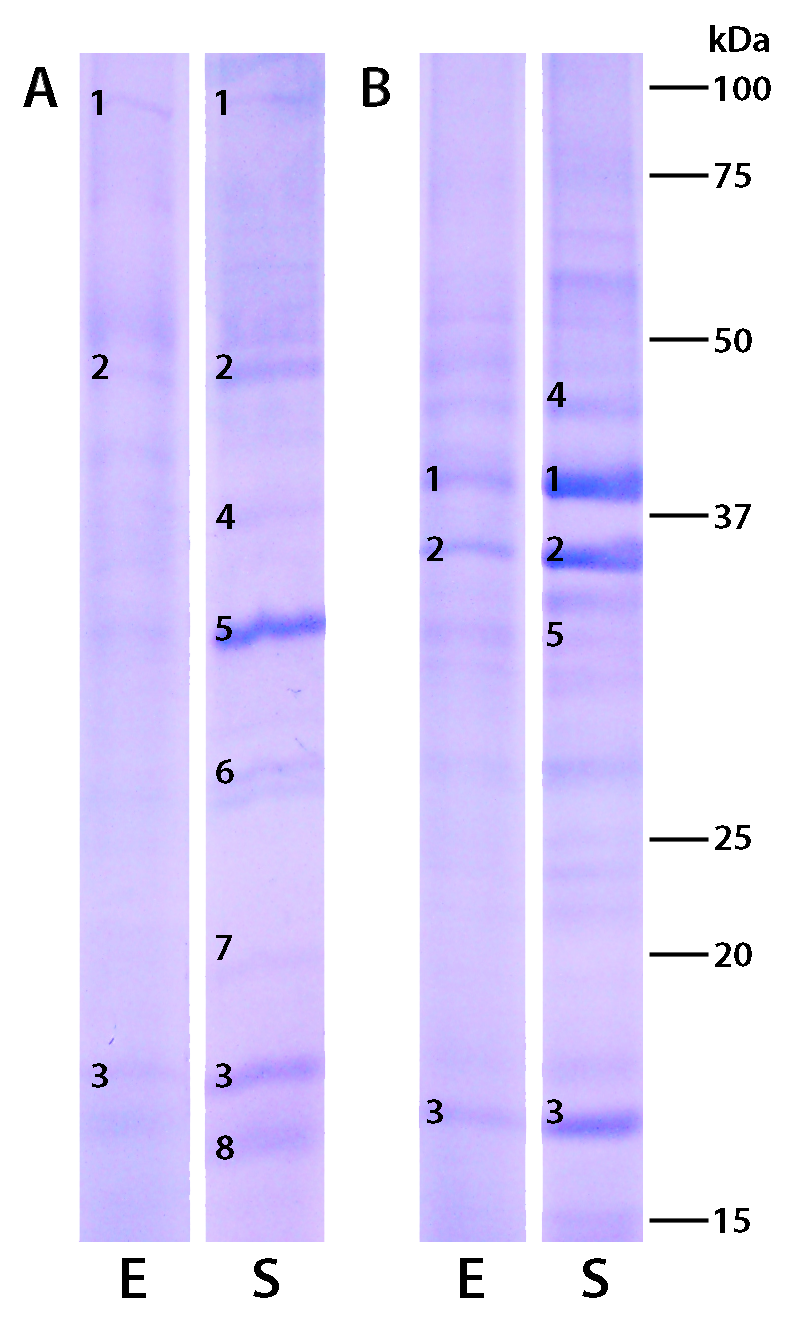

Supplement: Additional file 6 — Secreted proteins of P. avidum and P. granulosum. A) P. avidum ATCC25577 and B) P. granulosum DSM20700 were grown in BHI medium to exponential (E) and early stationary (S) phase. Secreted proteins were precipitated from culture supernatants and separated on a SDS-PAGE gel (12%). Abundant bands (numbered) were subjected to MS identification (see Additional file 7 for all identified proteins). Under the applied growth conditions, the most abundantly secreted proteins of P. avidum and P. granulosum are a triacylglycerol lipase (band 5 in section A) and two lysophospholipases (bands 1 and 2 in section B), respectively. [file 1471-2164-14-640-S6.tiff]
